# Dynapenic abdominal obesity is associated with mild cognitive impairment in patients with cardiometabolic disease: a cross-sectional study

**DOI:** 10.1186/s12877-022-02948-1

**Published:** 2022-03-28

**Authors:** Kazuhito Oba, Yoshiaki Tamura, Joji Ishikawa, Hiroyuki Suzuki, Yoshinori Fujiwara, Aya Tachibana, Remi Kodera, Kenji Toyoshima, Yuko Chiba, Atsushi Araki

**Affiliations:** 1 Department of Diabetes, Metabolism, and Endocrinology, Tokyo Metropolitan Geriatric Hospital, 35-2 Sakae-cho, Itabashi-ku, Tokyo, 173-0015 Japan; 2 Department of Cardiology, Tokyo Metropolitan Geriatric Hospital, Tokyo, Japan; 3 Research Team for Social Participation and Community Health, Tokyo Metropolitan Institute of Gerontology, Tokyo, Japan

**Keywords:** Dynapenic abdominal obesity, Mild cognitive impairment, Handgrip strength, Waist circumference, Dynapenia, Cardiometabolic disease

## Abstract

**Background:**

Dementia is an important health issue for older people and requires early intervention in the mild cognitive impairment (MCI) stage to manage risk factors. Both dynapenia (DP) and abdominal obesity (AO) are associated with inflammation and oxidative stress, which may be involved in the pathogenesis of cognitive impairment. Therefore, in this cross-sectional study, we aimed to evaluate the association between MCI and dynapenic abdominal obesity (DAO), a combination of DP and AO.

**Methods:**

A total of 417 older outpatients with cardiometabolic diseases without severe cognitive impairment were studied to compare cognitive function in four groups: control, DP, AO, and DAO groups. DAO was defined as the combination of DP (handgrip strength of < 28 kg and < 18 kg in men and women, respectively) and AO (waist circumference of ≥ 85 cm and ≥ 90 cm in men and women, respectively). MCI was defined as a score of ≤ 25 in the Japanese version of the Montreal Cognitive Assessment. Multiple regression analyses were performed to examine if MCI was independently associated with DAO, low handgrip strength, or high waist circumference.

**Results:**

The DAO group obtained the lowest cognitive test scores and had the highest prevalence of MCI. Furthermore, after adjusting for covariates, the logistic regression analysis showed that patients in the DAO group were at an increased risk of MCI (odds ratio [OR] = 3.98, 95% confidence interval [CI]: 1.15–13.77). Further logistic regression analyses revealed that both low handgrip strength (OR = 2.19, 95% CI: 1.11–4.29) and high waist circumference (OR = 2.03, 95% CI: 1.03–3.99) were associated with MCI.

**Conclusions:**

DAO, which can be easily diagnosed by a combination of handgrip strength and waist circumference, was associated with MCI in patents with cardiometabolic metabolic disease. This study suggests that screening for MCI in DAO patients could be important for early intervention of dementia prevention.

**Supplementary Information:**

The online version contains supplementary material available at 10.1186/s12877-022-02948-1.

## Background

Dementia is a significant health concern in older people, causing reduced quality of life, infections, eating problems, and high mortality [1]. Early interventions during the stage of mild cognitive impairment (MCI) are necessary to manage modifiable risk factors including diabetes mellitus (DM), physical inactivity, and social isolation [2–4]. In addition to DM, persons with cognitive dysfunction are associated with cardiovascular disease, metabolic syndrome [5], and poor muscle function [6]. Dynapenia (DP) is defined as the age-related decline in muscle strength; it is different from sarcopenia as the latter focuses on low muscle mass [7]. Additionally, previous studies have found an association between DP and cognitive decline [6, 8].

Compared to middle-aged people, obesity reduces the risk of dementia in older people [9–11]; however, abdominal obesity (AO) increases the risk of developing cognitive impairment and dementia, regardless of body mass index (BMI) level, even in older people [12, 13].

Cognitive dysfunction, DP, and AO have been reported to be associated with common pathological aetiologies, including inflammation [14–17], oxidative stress [18–20], and insulin resistance [21–23]. Malnutrition is also related to cognitive decline and DP [24, 25]. Sequentially, these aetiologies are associated with cardiometabolic disease [26] and aging [27]. Therefore, it is hypothesised that dynapenic AO (DAO), the combination of DP and AO, increases the risk of cognitive dysfunction. However, studies demonstrating the relationship between DAO and MCI or cognitive impairment remain lacking. Therefore, our study aimed to examine the association between DAO and MCI in older patients with cardiometabolic disease in a cross-sectional study. Additionally, we investigated whether DP and AO were independently related to MCI.

## Methods

### Subjects

**Fig. 1:**
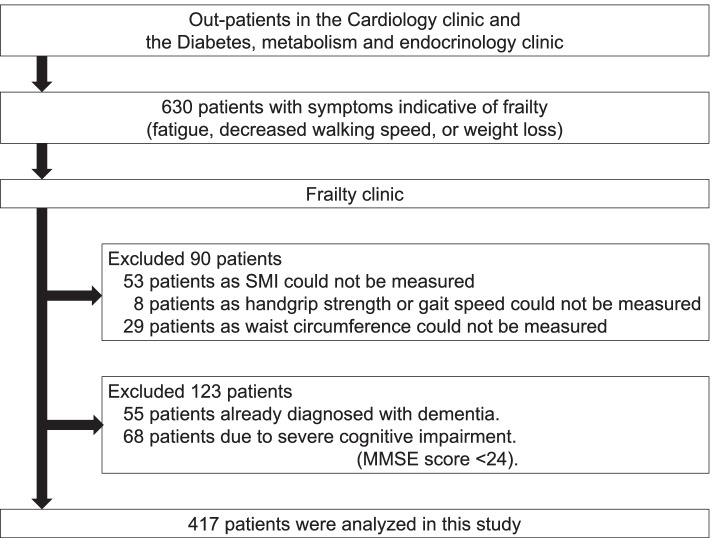
Flow chart of the recruitment of subjects. MMSE: Mini-Mental State Examination

This study included 630 consecutive outpatients who visited the frailty clinic between October 2015 and November 2020 [28]. Among them, patients with symptoms indicative of frailty (fatigue, decreased walking speed, or weight loss) were recruited from outpatients with regular hospital visits at the cardiology and the diabetes, metabolism and endocrinology clinics in the Tokyo Metropolitan Geriatric Hospital. Among the 630 outpatients, 90 patients could not undergo the measurement of waist circumference, handgrip strength, lower extremity muscle mass or gait speed. Additionally, we excluded 123 patients with severe cognitive impairment, a Mini-Mental State Examination (MMSE) score of < 24, or history of dementia diagnosis. Finally, 417 older patients were included in the analysis (Fig. 1).

### Assessment and definition of AO

Waist circumference was measured at the frailty clinic. Body fat percentage was calculated as the quotient of the body fat amount (kg) and body weight (kg). The amount of body fat was measured using the bioelectrical impedance analysis (BIA) method using InBody 770® (InBody Japan Inc., Tokyo) [28].

AO was defined as a high waist circumference (≥ 85 cm in men and ≥ 90 cm in women) according to the criteria for metabolic syndrome in Japan [29]. Obesity was defined as body fat percentage above the 60th percentile of this study [30], which was ≥ 28.0% for men and ≥ 34.7% for women.

### Assessments and definition of DP and sarcopenia

The handgrip strength on both sides were measured twice using a dynamometer (Takei Scientific Instruments, Niigata, Japan), and the maximum of the four measurements was adopted. The appendicular skeletal muscle index (ASMI) was calculated as the quotient of the appendicular muscle mass (kg) and the square of height (m^2^). The appendicular muscle mass was measured by the BIA method using InBody 770®. Gait speed (m/s) was calculated using the time in the middle 4 m of a 6 m walk [28].

DP (i.e., low muscle strength) was defined as a handgrip strength of < 28 kg for men and < 18 kg for women. Sarcopenia (i.e., low ASMI) was defined as ASMI of < 7.0 kg/m^2^ for men and < 5.7 kg/m^2^ for women. Low gait speed was defined as a gait speed of < 1.0 m/s [31].

### Definition of dynapenic AO or sarcopenic obesity

DAO was defined as the combination of DP and AO, as described above. According to the presence or absence of DP or AO, all subjects were divided into four DAO category groups: DAO group (*n* = 93); DP alone group (DP), (*n* = 113); AO alone group (AO) (*n* = 111); and control group (C) (*n* = 100).

Furthermore, according to the presence or absence of sarcopenia (low ASMI) or obesity, we classified the subjects into four SO categories groups: sarcopenic obesity groups (SO) (*n* = 65); sarcopenia alone group (S) (*n* = 128); obesity alone group (O) (*n* = 103); and control group (C) (*n* = 121).

### Cognitive function assessment and definition of MCI

The MMSE, Japanese version of the Montreal Cognitive Assessment (MoCA-J) [32], and Hasegawa’s Dementia Scale-Revised (HDS-R) were used to assess cognitive function [28]. MCI was defined as MoCA-J score ≤ 25, which has been previously reported to have a sensitivity of 93.0% and specificity of 87.0% for the diagnosis of MCI in Japanese subjects [32].

### Laboratory examination

All subjects underwent blood sampling to measure serum albumin, plasma glycohaemoglobin (HbA1c), serum low density lipoprotein (LDL)-cholesterol, serum high density lipoprotein (HDL)-cholesterol, serum triglyceride (TG), serum creatinine, serum high-sensitivity C-reactive protein (hsCRP), and plasma brain natriuretic peptide (BNP) levels. The estimated glomerular filtration rate (eGFR) was calculated using the serum creatinine level, age, and sex [33].

### Other evaluations

Information regarding the educational background and the history of DM, hypertension (HT), dyslipidaemia, ischemic heart disease (IHD), and stroke diagnoses were obtained from medical records. Blood pressure was measured on the left arm in a sitting position. Depressed mood was assessed using the Japanese version of the Geriatric Depression Scale 15 (GDS-15-J) [34]. Nutritional status was assessed using the Mini-Nutritional Assessment-Short Form (MNA-SF) [35]. Additionally, using the MNA-SF, we assessed decreased food intake and weight loss over a 3-month period using a questionnaire on food intake and weight loss (at least 3 kg).

### Statistical analysis

Data are described as the mean ± SD or frequency with percentages.　Differences in categorical and continuous variables were analysed using chi-square test and one-way analysis of variance (ANOVA), respectively.

Using MCI as the objective variable, three types of multiple logistic regression analyses were performed: (1) The odds ratios (ORs) and 95% confidence intervals (CIs) of MCI in the DAO, DP, and AO groups of the DAO category were calculated using the control group as a reference. Setting the following explanatory variables: (2) Without classifying the DAO groups, the independent association of MCI with low handgrip strength and high abdominal circumference were analysed, after adjusting for covariates. (3) Without using the SO categories, the independent association of MCI with low ASMI and obesity, defined as body fat percentage were analysed, after adjusting for covariates.

Multiple logistic regression analyses were performed using the following models: crude model (model 1), model adjusted for age, sex, education, and GDS-15-J score (model 2), and model further adjusted for systolic blood pressure, serum albumin, plasma HbA1c, serum LDL and HDL cholesterol, serum TG levels, eGFR, and plasma BNP levels (model 3). The above model was further adjusted for nutritional factors including decreased food intake (model 4) and weight loss (model 5) during the 3-month period.

Next, multiple linear regression analysis was performed using the MoCA-J score as the objective variable and handgrip strength, waist circumference, age, sex, education, and GDS-15-J score as explanatory variables.

All statistical analyses were performed using "SPSS Statistics 20" software package (IBM, Armonk, NY, USA). For all comparisons, statistical significance was set at *P* < 0.05.

## Results

### Clinical characteristics of all patients and subjects by DAO category

The clinical characteristics of all patients and the subjects classified by the DAO category are shown in Table 1. The mean age of patients was 78.3 years, ranging from 50 to 96 years, and 97.6% of patients were over the age of 65 years.

**Table 1:** Clinical characteristics in all patients and the DAO category groups

	All patients (*n* = 417)	DAO category groups				*P*-value
		C (*n* = 100)	AO (*n* = 111)	DP (*n* = 113)	DAO (*n* = 93)	
Age	78.3 ± 6.7	76.1 ± 6.7	76.1 ± 6.4	80.8 ± 5.8^*♰^	80.2 ± 6.4^*♰^	< 0.001^§^
Male (%)	35.5	25.0	45.9^*^	24.8^♰^	47.3^*‡^	< 0.001^§^
BMI (kg/m^2^)	23.4 ± 3.7	21.2 ± 2.2	26.0 ± 2.8^*^	20.9 ± 3.0^♰^	25.8 ± 3.0^*‡^	< 0.001^§^
MNA-SF score	11.1 ± 2.4	10.2 ± 2.6	12.5 ± 1.7^*^	10.2 ± 2.3^♰^	11.6 ± 2.2^*♰‡^	< 0.001^§^
Education (years)	12.2 ± 2.6	12.6 ± 2.3	13.0 ± 2.7	11.7 ± 2.6^♰^	11.5 ± 2.6^*♰^	< 0.001^§^
GDS-15-J score	4.7 ± 3.2	4.6 ± 3.3	3.9 ± 3.2	4.9 ± 2.9	5.4 ± 3.4^♰^	0.014^§^
Diabetes mellitus (%)	52.7	39.4	53.3	52.2	66.7^*^	0.002^§^
Hypertension (%)	75.9	67.7	85.0^*^	69.6^♰^	81.5	0.006^§^
Dyslipidemia (%)	65.4	62.2	70.6	58.4	71.0	0.141
Stroke (%)	9.2	4.0	10.1	5.3	18.3^*‡^	0.002^§^
Ischemic heart disease (%)	14.4	9.0	15.3	15.0	18.5	0.294
Systolic blood pressure (mmHg)	134 ± 19	132 ± 19	136 ± 17	133 ± 18	136 ± 20	0.185
Albumin (g/dl)	4.0 ± 0.3	4.0 ± 0.3	4.0 ± 0.3	3.8 ± 0.4^*♰^	3.9 ± 0.4^♰^	< 0.001^§^
HbA1c (%)	6.5 ± 0.9	6.3 ± 0.7	6.6 ± 1.0	6.4 ± 0.8	6.7 ± 0.9^*^	0.016^§^
LDL cholesterol (mg/dl)	110 ± 28	115 ± 29	111 ± 28	110 ± 26	105 ± 29	0.143
HDL cholesterol (mg/dl)	59 ± 15	66 ± 18	54 ± 14^*^	61 ± 14^♰^	53 ± 12^*‡^	< 0.001^§^
TG (mg/dl)	132 ± 72	115 ± 57	145 ± 70^*^	117 ± 58^♰^	152 ± 94^*‡^	< 0.001^§^
eGFR (ml/min)	58.3 ± 17.3	59.9 ± 15.9	57.2 ± 15.0	58.3 ± 17.7	57.9 ± 20.7	0.730
hsCRP (mg/dl)	0.23 ± 0.75	0.10 ± 0.16	0.15 ± 0.19	0.29 ± 1.04	0.39 ± 1.09	0.059
BNP (pg/ml)	66.0 ± 97.7	43.7 ± 44.7	58.2 ± 92.6	82.9 ± 135.7^*^	79.8 ± 87.5	0.019^§^
Handgrip strength (male) (kg)	27.9 ± 6.4	32.1 ± 3.6	33.2 ± 4.0	23.5 ± 3.6^*♰^	22.3 ± 4.2^*♰^	< 0.001^§^
Handgrip strength (female) (kg)	18.0 ± 4.5	21.7 ± 2.6	21.5 ± 3.2	14.4 ± 2.7^*♰^	14.6 ± 2.5^*♰^	< 0.001^§^
ASMI (male) (kg/m^2^)	7.17 ± 0.80	6.92 ± 0.95	7.65 ± 0.57^*^	6.61 ± 0.54^♰^	7.12 ± 0.76^♰‡^	< 0.001^§^
ASMI (female) (kg/m^2^)	5.82 ± 0.81	5.73 ± 0.52	6.37 ± 0.74^*^	5.34 ± 0.84^*♰^	6.12 ± 0.69^*‡^	< 0.001^§^
Sarcopenia (%)	46.3	45.0	18.9^*^	77.9^*♰^	41.9^♰‡^	< 0.001^§^
Gait speed (m/s)	1.12 ± 0.30	1.26 ± 0.29	1.17 ± 0.27	1.03 ± 0.30^*♰^	1.01 ± 0.28^*♰^	< 0.001^§^
Slow gait speed (%)	30.8	15.2	26.1	42.9^*^	38.9^*^	< 0.001^§^
Waist circumference (male) (cm)	88.3 ± 8.1	80.2 ± 3.7	94.0 ± 6.2^*^	79.7 ± 3.3^♰^	91.7 ± 5.3^*‡^	< 0.001^§^
Waist circumference (female) (cm)	87.5 ± 11.4	80.0 ± 6.5	98.6 ± 7.8^*^	80.2 ± 6.8^♰^	98.0 ± 7.5^*^	< 0.001^§^
Body-fat percentage (male) (%)	26.5 ± 6.2	22.5 ± 6.6	27.8 ± 5.6^*^	22.8 ± 5.7^♰^	29.8 ± 4.5^*‡^	< 0.001^§^
Body-fat percentage (female) (%)	32.0 ± 9.2	27.4 ± 7.0	38.6 ± 6.1^*^	27.1 ± 8.4^♰^	39.4 ± 6.1^*‡^	< 0.001^§^

*C* Control group, *AO* Abdominal Obesity group, *DP* Dynapenia group, *DAO* Dynapenic Abdominal Obesity group, *BMI* Body mass index, *GDS-15-J* Japanese version of the Geriatric Depression Scale 15, *HbA1c* Glycohemoglobin, *LDL* Low-density lipoprotein, *HDL* High-density lipoprotein, *TG* Triglyceride, *eGFR* Estimated glomerular filtration rate, *hsCRP* High-sensitivity C-reactive protein, *BNP* Brain natriuretic peptide, *ASMI* Appendicular skeletal muscle index

^*^ *p* < 0.05 (vs C), ♰ *p* < 0.05 (vs AO), ‡ *p* < 0.05 (vs DP), § *p* < 0.05 (ANOVA)

Subjects in the DAO and DP groups were older compared to the AO and C groups, the DAO and AO groups had a higher prevalence of males.

Handgrip strength and gait speed were lower in the DAO and DP groups compared to the AO and C groups. In both sexes, the AO group demonstrated the highest ASMI, followed by the DAO, C, and DP groups; in contrast, this order was reversed for the prevalence of sarcopenia. The DAO and AO groups had higher BMI, waist circumference, and body fat percentage compared to the DP and C groups. The DAO and AO groups had had higher scores on the MNA-SF than the C and DP groups.

### Cognitive function and prevalence of MCI in groups of DAO and SO categories

**Fig. 2:**
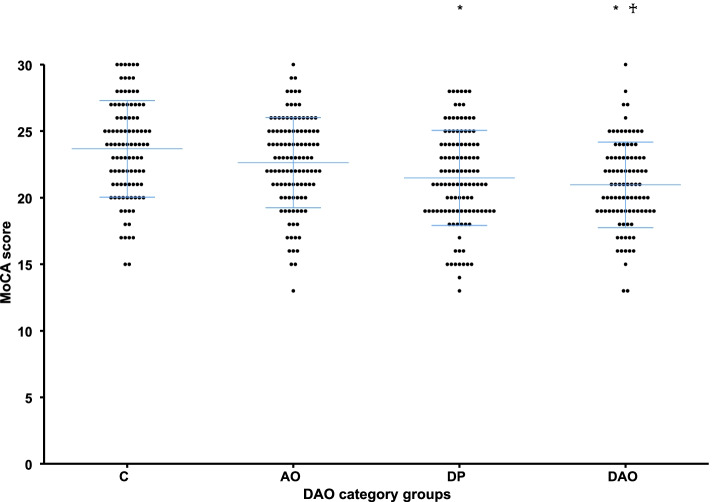
Comparison of MoCA-J scores in DAO category groups. C: control group, AO: abdominal obesity group. DP: dynapenia group, DAO: dynapenic　abdominal obesity group, MoCA-J: Japanese version of Montreal cognitive Assessment. * *p* < 0.001 (vs C), ♰ *p* < 0.01 (vs AO) (Bonferroni test). The middle bar is the mean value, and the error bars are the standard deviation

The cognitive functions (HDS-R, MMSE, and MoCA-J score) in the DAO category groups are shown in Table 2 and the scatter plot of MoCA-J scores is depicted in Fig. 2.　The DAO group had the lowest scores, and the DP group had the second lowest HDS-R, MMSE, and MoCA-J scores. Significant difference was observed between the DAO, DP, and C groups. The DAO group had significantly lower HDS-R and MoCA-J scores compared to the AO group. The prevalence of MCI in the DAO group was the highest at 94.6%, followed by 85.0% in the DP group, 79.3% in the AO group, and 69.0% in the C group; it was significantly higher in the DAO group than in the AO and C groups (*p* < 0.05 and *p* < 0.001, respectively). The DP group had a significantly higher prevalence of MCI than the C group (*p* < 0.05).

**Table 2:** Cognitive function in DAO category groups

	All patients (*n* = 417)	DAO category groups				*P*-value
		C (*n* = 100)	AO (*n* = 111)	DP (*n* = 113)	DAO (*n* = 93)	
HDS-R score	26.7 ± 2.9	27.8 ± 2.1	26.9 ± 2.7	26.5 ± 3.0^*^	25.6 ± 3.1^*♰^	< 0.001^§^
MMSE score	28.1 ± 1.7	28.6 ± 1.4	28.1 ± 1.7	28.0 ± 1.7^*^	27.6 ± 1.7^*^	< 0.001^|§^
MoCA-J score	22.2 ± 3.6	23.7 ± 3.6	22.6 ± 3.4	21.5 ± 3.6^*^	21.0 ± 3.2^*♰^	< 0.001^§^

*C* Control group, *AO* Abdominal Obesity group, *DP* Dynapenia group, *DAO* Dynapenic Abdominal Obesity group, *HDS-R* Hasegawa’s Dementia Scale-Revised, *MMSE* Mini-Mental State Examination, *MoCA-J* Japanese version of Montreal Cognitive Assessment

^*^ *p* < 0.05 vs C, ♰ *p* < 0.05 vs AO, ‡ *p* < 0.05 vs DP,§ *p* < 0.05 (ANOVA)

In contrast, no significant differences were observed between the cognitive function scores of the four groups of the SO category (Supplementary Table 1).

### Logistic regression analysis for the association between DAO category and MCI

The ORs and 95% CIs of MCI in the DAO, DP, and AO groups of the DAO category are shown in Table 3 (model 1–3) and Supplemental Table 2 (model 4 and 5). In the crude model 1, the DAO and DP groups were significantly associated with MCI (OR = 7.91, 95% CI: 2.92–21.4 and OR = 2.54, 95%CI: 1.30- 4.95, respectively); however, only the DAO group retained this relationship in the adjusted models 2 (OR = 4.29, 95% CI: 1.37–13.47) and 3 (OR = 3.98, 95% CI: 1.15–13.77) (Table 3). Furthermore, there was a significant association between DAO and MCI even after adjusting for nutritional factors (decreased food intake or weight loss) (models 4 and 5) (Supplemental Table 2).

**Table 3:** Odds ratios for MCI in the groups of DAO category in multiple logistic regression analysis

	C OR (95% CI)	AO OR (95% CI)	DP OR (95% CI)	DAO OR (95% CI)
Model 1	1 (reference)	1.72 (0.92- 3.21)	2.54 (1.30- 4.95)	7.91 (2.92–21.40)
Model 2	1 (reference)	1.26 (0.62- 2.55)	1.31 (0.63- 2.77)	4.29 (1.37–13.47)
Model 3	1 (reference)	1.50 (0.66- 3.41)	1.47 (0.63- 3.43)	3.98 (1.15–13.77)

Model 1: crude model

Model 2: adjusted for age, sex, education, and GDS-15-J score

Model 3: further adjusted for Systolic blood pressure, serum albumin, plasma HbA1c, serum LDL cholesterol, serum HDL cholesterol, and serum TG, stroke, eGFR, and plasma BNP

*MCI* Mild Cognitive Impairment (defined as low MoCA-J score of ≤ 25), *OR* Odds Ratio, *CI* Confidence Interval

### Multiple logistic or linear regression analysis for the association between MCI and handgrip strength or waist circumference

Multiple logistic regression analysis was used to determine if MCI was independently associated with low handgrip strength and high waist circumference. In models 1–3, low handgrip strength and high waist circumference were independently associated with MCI (Table 4). In model 3, the ORs of MCI with low handgrip strength and high waist circumference were 2.19 and 2.03, respectively. Multiple linear regression analysis after adjustment for age, sex, education, and GDS-15-J score also revealed that handgrip strength and waist circumference were independently associated with MoCA-J score (standardized partial regression coefficients: β = 0.20, *p* = 0.004 and β = -0.08, *p* = 0.074, respectively).

**Table 4:** Association between low handgrip strength or high waist circumference and MCI in the logistic regression analysis

	Low handgrip strength OR (95% CI)	High waist circumference OR (95% CI)
Model 1	3.09 (1.79–5.34)	2.04 (1.20–3.45)
Model 2	2.45 (1.38–4.37)	2.17 (1.25–3.78)
Model 3	2.19 (1.11–4.29)	2.03 (1.03–3.99)

Model 1: crude model

Model 2: adjusted for age, sex, education, and GDS-15-J score

Model 3: further adjusted for Systolic blood pressure, serum albumin, plasma HbA1c, serum LDL cholesterol, serum HDL cholesterol, and serum TG, stroke, eGFR, and plasma BNP

*MCI* Mild Cognitive Impairment (defined as low MoCA-J score of ≤ 25), *OR* Odds Ratio, *CI* Confidence Interval

On the other hand, when sarcopenia (low ASMI) and obesity (high body fat percentage) were entered as explanatory variables, no significant associations were observed between these variables and MCI (data not shown).

## Discussion

This study showed that DAO was associated with MCI even after adjusting for covariates in patients with cardiometabolic disease. This is the first study to show an independent association between DAO and cognitive impairment. We also found low handgrip strength and high waist circumference, the components of DAO, were independently associated with MCI. On the other hand, no relationship was observed between a low ASMI or body fat percentage and MCI. Our results suggest that muscle weakness and AO are more associated with MCI, compared with muscle loss and simple obesity.

These findings are consistent with previous reports that separately analysed the relationship of cognitive function with muscle strength or waist circumference [6, 8, 12, 36]. One study in Taiwan found that low muscle strength and low physical performance were more associated with global cognitive function impairment, compared to sarcopenia [8]. A longitudinal study demonstrated that poor muscle function is a better predictor of incidence MCI and cognitive decline, compared to reduced lean muscle mass [6]. On the other hand, it has been reported that despite normal BMI, high waist circumference increases the risk of developing dementia in older people [12]. Additionally, central fat mass was associated with MCI, but skeletal muscle mass was not [36]. These data suggest that DP with AO potentially contribute to the development of MCI. The absence of an interaction suggests that these two variables additively influence cognitive impairment.

The association between DAO and MCI might be explained by nutritional factors. Weight loss or malnutrition may predict cognitive decline and muscle weakness (which may lead to DAO) even in obese patients [24, 37]. However, the association between DAO and cognitive dysfunction persisted even after adjusting for weight loss and decreased dietary intake, suggesting the unlikely involvement of malnutrition in our study.

The mechanism of the association between DAO and cognitive impairment is unknown; however, it may be mediated by insulin resistance and inflammation [38]. A previous study showed the relationship between DAO and elevated inflammatory markers [39]. Furthermore, some reports have shown that DP is associated with markers of oxidative stress, inflammation, insulin resistance [40], and mitochondrial disease [41]. On the other hand, several studies suggest that abdominal or visceral obesity may contribute to cognitive impairment via inflammatory cytokines [42, 43], cerebral microangiopathy, and cerebral white matter lesions [43, 44].

DAO is a significant health concern for older people, since it has been associated with decreased physical function [45], decreased activities of daily living [46], increased occurrence of falls [47], increased mortality [48], and MCI. Therefore, early intervention for DAO is essential in patients with cardiometabolic diseases. Assessing MCI in patients with DAO is an important aspect of dementia prevention; especially since it can be diagnosed using only two simple measurements: handgrip strength and waist circumference. The combination of resistance exercise, energy restriction, and cognitive training might be important measures for preventing dementia in patients with DAO.

This study has several limitations. First, since this was a cross-sectional study, it was not possible to determine the causal relationships. Second, most participants were patients with cardiometabolic diseases such as DM, HT, and dyslipidaemia, who visited a single hospital in Japan; therefore, the results of this study should be confirmed in other institutions and other population-based studies. Third, the diagnosis of MCI is comprehensively established using history, cognitive screening tests, blood tests, and diagnostic criteria, such as Clinical Dementia Rating and the Diagnostic and Statistical Manual version 5; Our study using the MoCA-J for detecting MCI may potentially overestimate or underestimate the diagnosis of MCI. However, a recent meta-analysis showed that the MOCA is a more effective screening test than MMSE to detect MCI [49]. Although the diagnostic accuracy of MoCA is comparable to that of the Consortium to Establish a Registry for Alzheimer’s Disease (CERAD) and Quick Mild Cognitive Impairment (Qmci) [50], our previous study showed that MoCA-J had excellent sensitivity and specificity in diagnosing MCI when the cut-off value similar to this study was used [32]. Therefore, it is useful to use the well-validated MoCA-J for screening of MCI and take measures for prevention of cognitive decline in clinical practice. Finally, other confounding factors such as nutritional status, physical activity, inflammatory markers, insulin resistance, oxidative stress markers, and mitochondrial function, may influence the association between DAO and MCI; however, these were not analysed in this study.

In conclusion, DAO, defined as the combination of decreased handgrip strength and high waist circumference, is associated with MCI in older patients with cardiometabolic diseases. Our results may suggest that screening for MCI in DAO patients could be important for early intervention of dementia prevention. Further longitudinal studies are needed to examine the effectiveness of DAO in predicting cognitive decline or the incidence of MCI and dementia.

## Supplementary Information

**Additional file 1.**

## Data Availability

The data that support the findings of this study are available on request from the corresponding author. The data are not publicly available on information that could compromise the privacy of the patient.

## Abbreviations

DAO: Dynapenic abdominal obesity
DP: Dynapenia
AO: Abdominal obesity
MoCA-J: Japanese version of the Montreal Cognitive Assessment
MCI: Mild cognitive impairment
MMSE: Mini mental state examination
HDS-R: Hasegawa's Dementia Scale-Revised
HbA1c: Haemoglobin A1c
HDL: High density lipoprotein
LDL: Low density lipoprotein
TG: Triglycerides
hsCRP: High sensitivity C-reactive protein
BNP: Brain natriuretic peptide
eGFR: Estimated glomerular filtration rate
DM: Diabetes mellitus
ASMI: Appendicular skeletal muscle index
