# Supplementary material for: Dynapenic abdominal obesity is associated with mild cognitive impairment in patients with cardiometabolic disease: a cross-sectional study
Source: BMC Geriatr. 2022 Mar 28;22:255. doi: 10.1186/s12877-022-02948-1 (PMC8962154; doi:10.1186/s12877-022-02948-1)
Supplement: Supplementary file 1 — Additional file 1. [file 12877_2022_2948_MOESM1_ESM.docx]

**Supplementary Table 1 Cognitive function in groups of SO category**

|  | All patients  (n=417) | Groups concerning sarcopenic obesity | | | |  |
| --- | --- | --- | --- | --- | --- | --- |
|  |  | C  (n=121) | O  (n=103) | S  (n=128) | SO  (n= 65) | p value |
| HDS-R score | 26.7 ± 2.9 | 26.7 ± 3.1 | 26.7 ± 2.4 | 26.9 ± 2.8 | 26.4 ± 3.2 | 0.702 |
| MMSE score | 28.1 ± 1.7 | 28.2 ± 1.8 | 27.8 ± 1.6 | 28.2 ± 1.7 | 28.0 ± 1.6 | 0.199 |
| MoCA-J score | 22.2 ± 3.6 | 22.6 ± 3.7 | 21.9 ± 3.3 | 22.3 ± 3.6 | 21.7 ± 3.8 | 0.267 |

C: control group: sarcopenia (-) and obesity (-), O: obesity group: sarcopenia (-) and obesity (+), S: sarcopenia group: sarcopenia (+) and obesity (-). SO: sarcopenic obesity group: sarcopenia (+) and obesity (+).

HDS-R: Hasegawa’s Dementia Scale-Revised, MMSE: Mini-Mental State Examination, MoCA-J: Japanese version of Montreal Cognitive Assessment.

**Supplementary Table 2 Odds ratio of MCI in groups of DAO categories in multiple logistic regression analysis after adjusted for nutritional factors**

|  | C  OR (95% CI) | AO  OR (95% CI) | DP  OR (95% CI) | DAO  OR (95% CI) |
| --- | --- | --- | --- | --- |
| Model 4 | 1 (reference) | 1.46 (0.64- 3.32) | 1.30 (0.57- 2.97) | 3.63 (1.06-12.47) |
| Model 5 | 1 (reference) | 1.55 (0.68- 3.52) | 1.28 (0.56- 2.91) | 4.00 (1.18-13.48) |

Model 4: adjusted for age, sex, education, GDS-15-J score, systolic blood pressure, serum albumin, plasma HbA1c, serum LDL cholesterol, serum HDL cholesterol, and serum TG, stroke, eGFR, plasma BNP, and decrease in food intake during the 3-month period.

Model 5: adjusted for age, sex, education, GDS-15-J score, systolic blood pressure, serum albumin, plasma HbA1c, serum LDL cholesterol, serum HDL cholesterol, and serum TG, stroke, eGFR, plasma BNP, and weight loss (≥3 kg) during the 3-month period.
